# Early emergence of inter-lobar heterogeneity in mouse embryonic lung

**DOI:** 10.1186/s13619-026-00303-y

**Published:** 2026-10-14

**Authors:** Yun Yang, Yonggao Fu, Shiting Li, Zhiwen Liu, Mingzhu Wen, Naihe Jing, Xianfa Yang

**Affiliations:** 1 Guangzhou Medical University, Guangzhou, Guangdong Province 511436 China; 2 Guangzhou National Laboratory, Guangzhou International Bio Island, No. 9 Xing Dao Huan Bei Road, Guangzhou, Guangdong Province 510005 China; 3 School of Life Science and Technology, Southeast University, Nanjing, 210096 China

**Keywords:** Lung development, Inter-lobar heterogeneity, Cell type composition, Spatial transcriptomics, gCap endothelial cells

## Abstract

The mammalian lung is organized into anatomically distinct lobes, yet whether regional heterogeneity arises among different lobes remains poorly understood. Here, we performed comprehensive transcriptomic profiling of individual lung lobes at the pseudoglandular stage. Comparative analyses revealed that consistent inter-lobar divergence occurs primarily at the level of protein-coding genes. Notably, the left lung lobe exhibits enrichment of immune-response-associated gene expression and increased abundance of early general capillary (gCap) endothelial cells, whereas the right cranial lobe preferentially expresses genes associated with ventricular cardiac muscle morphogenesis and shows reduced gCap enrichment. Integration with publicly available datasets and experimental validation further confirmed the heterogeneous distribution of gCap cells. Together, our findings indicate that molecular and cellular inter-lobar heterogeneity emerges during embryonic development and highlight the significance of considering lobe identity in understanding pulmonary development and related congenital diseases.

**Supplementary Information:**

The online version contains supplementary material available at https://doi.org/10.1186/s13619-026-00303-y.

## Background

The mammalian respiratory and cardiovascular systems are anatomically coordinated to ensure the circulation of oxygenated blood throughout the body, with the heart acting as the pump and the lung functioning as the oxygen-exchanging center (Krogh 2015; Yang et al. 2024). During embryogenesis, organ primordia start to interact with each other at the very beginning (Han et al. 2020; Ng et al. 2023), spatiotemporally co-develop (Miao et al. 2025; Peng et al. 2013), and ultimately establish a highly synchronized architectural and functional landscape. Notably, the lung usually forms more lobes and slightly larger lobe volume on the right than on the left, accommodating the left-side positioning of the heart. Beyond physical constraints, it has been reported that the spatial distribution of organ architectural units is related to the incidence of congenital diseases. For instance, the most commonly affected lobe in bronchial atresia is the left upper lobe, whereas congenital pulmonary airway malformations can be more frequently observed in the lower lung lobes (Durhan et al. 2021). The lobe-biased disease distributions suggest that cellular and molecular heterogeneity may arise during early organ development. However, the cellular and molecular basis underlying the physical architecture of the lung lobes remains largely unexplored.

In mice, lung development proceeds through five stages: embryonic, pseudoglandular, canalicular, saccular, and alveolar. The earliest lung primordium is specified within the foregut around embryonic day 9.0 (E9.0) to E9.5,(Yang et al. 2024) with the left and right anlagen forming independently. Then, each lung bud undergoes a repetitive process of outgrowth and branching, forming future airways during the pseudoglandular stage (E9.5-E16.5). By the end of this stage, the prototypical air-conducting bronchial tree up to the terminal bronchiole (16–20 generations) (Schittny 2017) and the major cell types blueprint (Negretti et al. 2021; Zepp et al. 2021) are established. The asynchronous cell type diversification features and the molecular dynamics of epithelial, endothelial, and mesenchymal lineages have been revealed (Negretti et al. 2021; Zepp et al. 2021). However, spatially resolved investigations of intra-organ architectural heterogeneity, particularly at the lobar level across the lung, are still limited.

In this study, we performed total transcriptomic sequencing, including both in-depth profiling of coding and noncoding expressed genes, on separated lung lobes from the E15.5 mouse lung, providing an integrated view of molecular divergence across each lobe during mammalian lung development. Through analyses with publicly available stage-matched single-cell and spatial transcriptomic datasets, we identified significant differences, subtle but non-negligible, among different lung lobes for the early formed lung. Specifically, the left lobe shows higher expression of immune response related genes and a significantly higher enrichment of early general capillary (gCap) cells, whereas the right cranial lobe is enriched with ventricular cardiac muscle morphogenesis genes and the lowest abundance of gCap cells, suggesting potential cross-organ interactions between pulmonary and cardiac tissues. These findings suggest that inter-lobar heterogeneity may contribute to spatial coordination among neighboring organs and potentially underlie lobe-biased congenital disease incidence. Collectively, our results highlight the importance of considering lung lobe-specific features in future developmental and disease studies and suggest that integrating developmental and clinical data will facilitate a deeper understanding of cross-organ interactions and related pathologies.

## Results

### Morphological tracking of mouse lung development using *Nkx2-1*-eGFP lineage tracing mouse

**Fig. 1:**
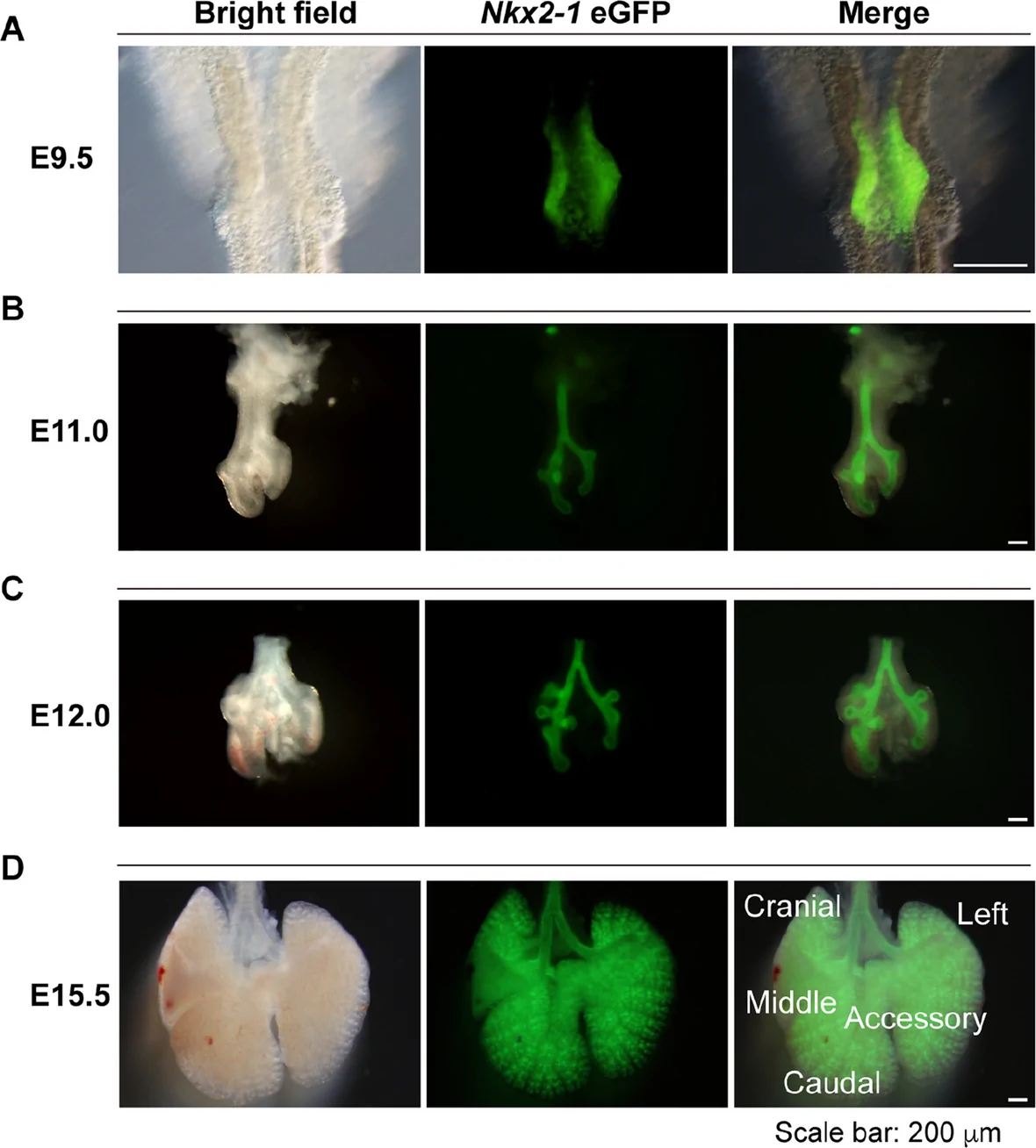
Morphological dynamics of mouse lung development. **A-D** Microscopic recording of lung primordium at representative embryonic stages (**A**: E9.5; **B**: E11.0; **C**: E12.0; **D**: E15.5). Expected lung primordia were dissected from mouse embryos at indicated stages. GFP signals precisely mark the location and morphological dynamics of lung epithelium. Scale bar: 200 μm

As reported, the lung epithelium emerges during early embryonic stages and subsequently undergoes a series of stereotyped branching morphogenetic events to establish the mature multi-lobar structure of the lung (Metzger et al. 2010). Specification of the lung epithelium, marked by the expression of *Nkx2-1*, from early foregut subgroups represents the onset of mouse lung development (Ikonomou et al. 2020). Here, to precisely track detailed morphological changes in mouse lung development, we employed the established *Nkx2-1-eGFP* lineage reporter mouse obtained from the Jackson Laboratory (Longmire et al. 2012). As shown, a localized GFP-positive endodermal diverticulum first emerges from the ventral wall of the anterior foregut (Fig. 1A). The primitive lung buds then bulge outwards into the surrounding pleuroperitoneal canals, with the two primary bilateral bronchi forming separately (Fig. 1B). By E12.0, the rudimentary lobar organization of the unilobed left and quadrilobed right lungs becomes apparent (Fig. 1C). Thereafter, iterative branching morphogenesis continues through repeated domain branching and tip bifurcation, progressively producing a hierarchical epithelial tree structure. At E15.5, all five lung lobes are clearly discernible (one left, four right: cranial, middle, caudal, accessory), accompanied by numerous distal epithelial tips (Fig. 1D). At this stage, the well-organized epithelial tubules, surrounded by dense mesenchyme within each lobe, are beginning to widen and getting ready for future vascularization. Together, these data suggest that the E15.5 primitive lung represents an optimal stage for investigating the earliest potential inter-lobar heterogeneity.

### In-depth transcriptomic profiling of the primary mouse lung lobe

**Fig. 2:**
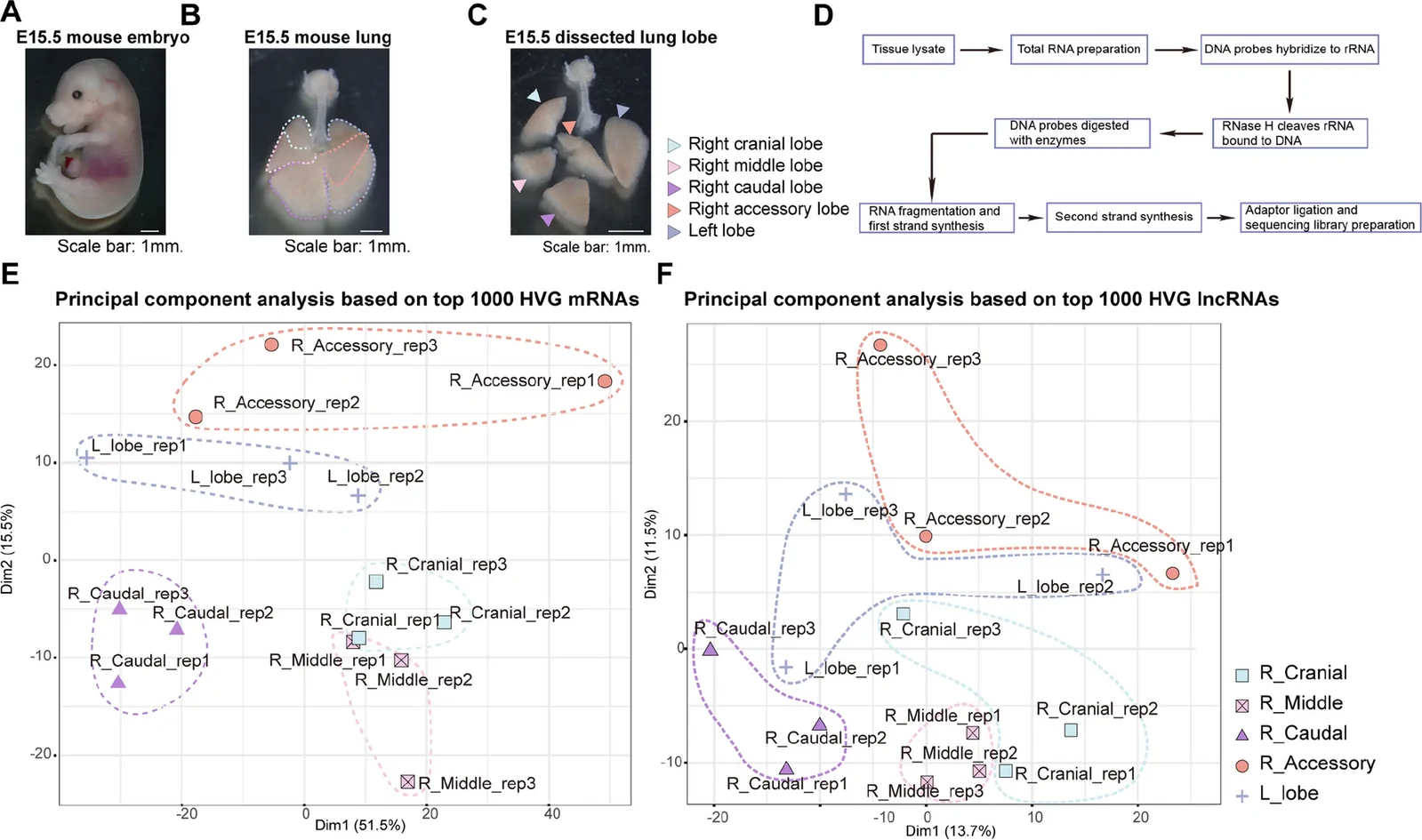
Whole transcriptome profiling for E15.5 lung lobes. **A-C** Morphology and dissection of E15.5 mouse lung. The lung tissue was first isolated from E15.5 mouse embryos (**A**), and then dissected based on the lung lobe separation (**B**), the residual attached tissues were carefully removed during this stage (**C**). **D** The schematic diagram showing the experimental diagram of library preparation for in-depth transcriptome sequencing of each lung lobe. Briefly, each lobe was first subjected to total RNA extraction, and then mammalian-specific ribosomal RNA probes were used to hybridize the ribosomal RNA and followed by RNase H cleavage; the rRNA-depleted lung lobe RNA was then subjected to standard transcriptome library preparation procedure for further detecting both polyadenylated and nonadenylated mRNA and non-coding RNAs. **E** Principal component analysis based on the top 1000 highly variable mRNA genes. Biological replicates for each lung lobe were marked with dotted colored circles. **F** Principal component analysis based on the top 1000 highly variable lncRNA genes. R_Cranial is short for right cranial lobe, R_Middle is short for right middle lobe, R_Caudal is short for right caudal lobe, R_Accessory is short for right accessory lobe, L_lobe is short for left lobe

To further resolve the molecular distinctions among individual lobes, we collected E15.5 mouse embryos and then carefully dissected the lung tissue and removed the residual surrounding non-lung tissues (Fig. 2A-B), the acquired lung tissue was then carefully released from the major bronchial tree and morphological intact right cranial, right middle, right caudal, right accessory, and left lobe were harvested (Fig. 2C). Following the standard workflow for ribosomal RNA (rRNA) depletion total RNA sequencing procedures, we first prepared the RNA samples from each lobe, and then subjected the acquired RNA samples to ribosomal RNA removal through specific DNA probe hybridization and digested with RNase H. The rRNA-depleted RNA samples were then subjected to RNA fragmentation and first cDNA strand synthesis, second cDNA strand synthesis, and subsequently sequencing library construction (Fig. 2D). For each lobe, three high-quality biological replicates were generated. As shown, an average of more than 16,000 mRNAs and 4,600 lncRNAs were detected per lobe (Fig. S1A-B). Biological replicates from each lobe also showed strong intra-group concordance, with pairwise correlation coefficients ranging from approximately 0.85 to 0.96 (Fig. S1C), demonstrating high reproducibility of the dataset. Thus, the deep transcriptomic coverage and high consistency among biological replicates establish a robust resource for exploring the key molecular features of each lobe.

### Integrative analyses revealing the presence of inter-lobar distinctions at both molecular and cellular levels

Due to deep transcriptomic coverage achieved in our datasets, we next tested for lobe-specific molecular signatures. As shown, to systematically determine molecular relationships among different lobes, we applied principal component analysis (PCA) to protein-coding and long non-coding gene dimensions, respectively. The top 1000 highly variable genes (HVGs) were selected as the PC loading genes. Interestingly, replicates for each lobe showed close clustering within the PCA plot but were separated from the other lobes within the PCA plot using the top 1000 HVG mRNAs (Fig. 2E). In contrast, when PCA was performed using the top 1,000 HVG lncRNAs, the samples became largely intermingled (Fig. 2F). These results suggest that substantial lobe-specific differences may exist at the level of coding-gene expression.

**Fig. 3:**
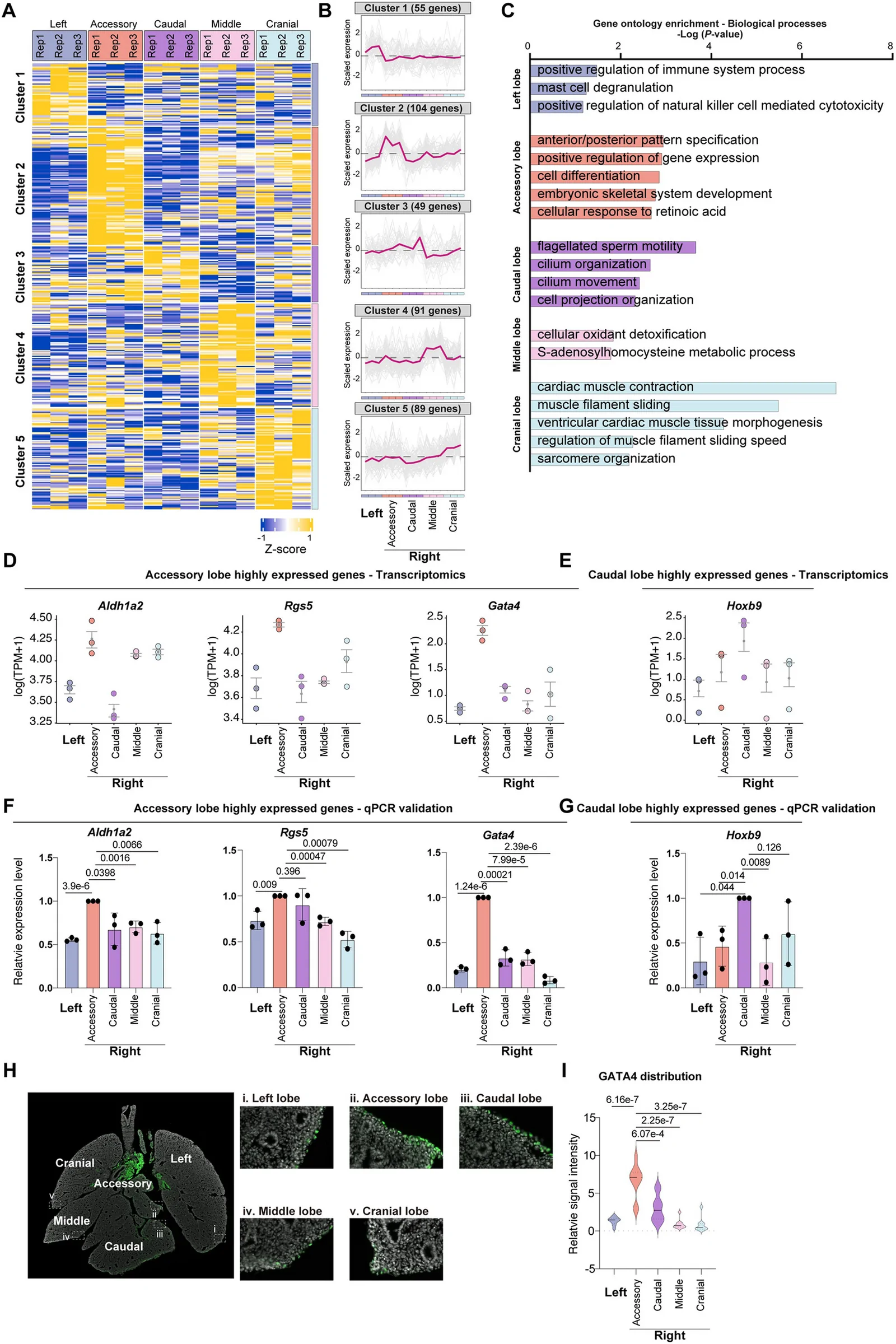
Lung lobe-specific transcriptomic architectures. **A** Heatmap showing the lobe-specific gene expression patterns. Differentially expressed genes were identified based on the criteria: adjusted *p*-value (FDR) < 0.05, an absolute log_2_(fold_change) > 0.5. **B** Line plots displaying the scaled expression level of lobe-specific genes. **C** Functional enrichment of lung lobe-specific genes. Gene ontology enrichment results for cluster 1, 3, 4, and 5 genes were shown. **D** The expression of *Aldh1a2*, *Rgs5*, and *Gata4* across lung lobes. **E** The expression of *Hoxb9* across lung lobes. **F** Quantitative PCR validating the relative expression of *Aldh1a2*, *Rgs5*, and *Gata4*. Student’s t-tests were used. **G** Quantitative PCR validating the relative expression of *Hoxb9*. Student’s t-test was used. **H** Immunofluorescence staining of GATA4 distribution in lung lobes. Representative snapshots of each lobe were labeled in dotted boxes and presented on the right. **I** Statistical summary of GATA4 staining signal. Student’s t-test was used

To determine lobe-specific molecular signatures at E15.5, we performed differentially expressed genes analysis and identified that each lobe displays enrichment of a specific gene module (Fig. 3A-B; Fig. S2A-B; Table S1). To specify, the left lobe shows higher expression of a set of 55 genes enriched for biological processes related to positive regulation of immune system processes (Fig. 3C). Interestingly, recent studies have reported that lipopolysaccharide (LPS) administration in mice induces a stronger inflammatory response in the left lobe, as evidenced by increased recruitment of IBA1-positive cells (Yoshida et al. 2026). Our observation here may suggest that lobe-specific immune predisposition is established during embryonic development, with the left lobe acquiring a distinct transcriptional program that could influence its differential sensitivity to inflammatory stimuli later in life. In contrast, we also found that the right cranial lobe shows higher expression of cardiac muscle contraction and ventricular cardiac muscle tissue morphogenesis related genes (Fig. 3C). Considering the close anatomical proximity between the right cranial lobe and the developing heart, potential cardiac tissue contamination was precluded by examining the expression of cardiomyocyte markers in the dissected lung lobes (Fig. S2C). Thus, the spatially restricted transcriptional program in the right cranial lobe may reflect shared morphogenetic cues that could contribute to coordinated cardiopulmonary development during embryogenesis, and raise the possibility that cardiopulmonary progenitor cells (Wen et al. 2025) may exhibit a preferential contribution to the right cranial lobe relative to the other lobes. Moreover, we also experimentally validated the distribution of lung-specific signatures (Fig. 3D-I). Consistent with the transcriptomic data, the mesothelial marker GATA4 showed obvious enrichment in the accessory lobe (Fig. 3H-I).

**Fig. 4:**
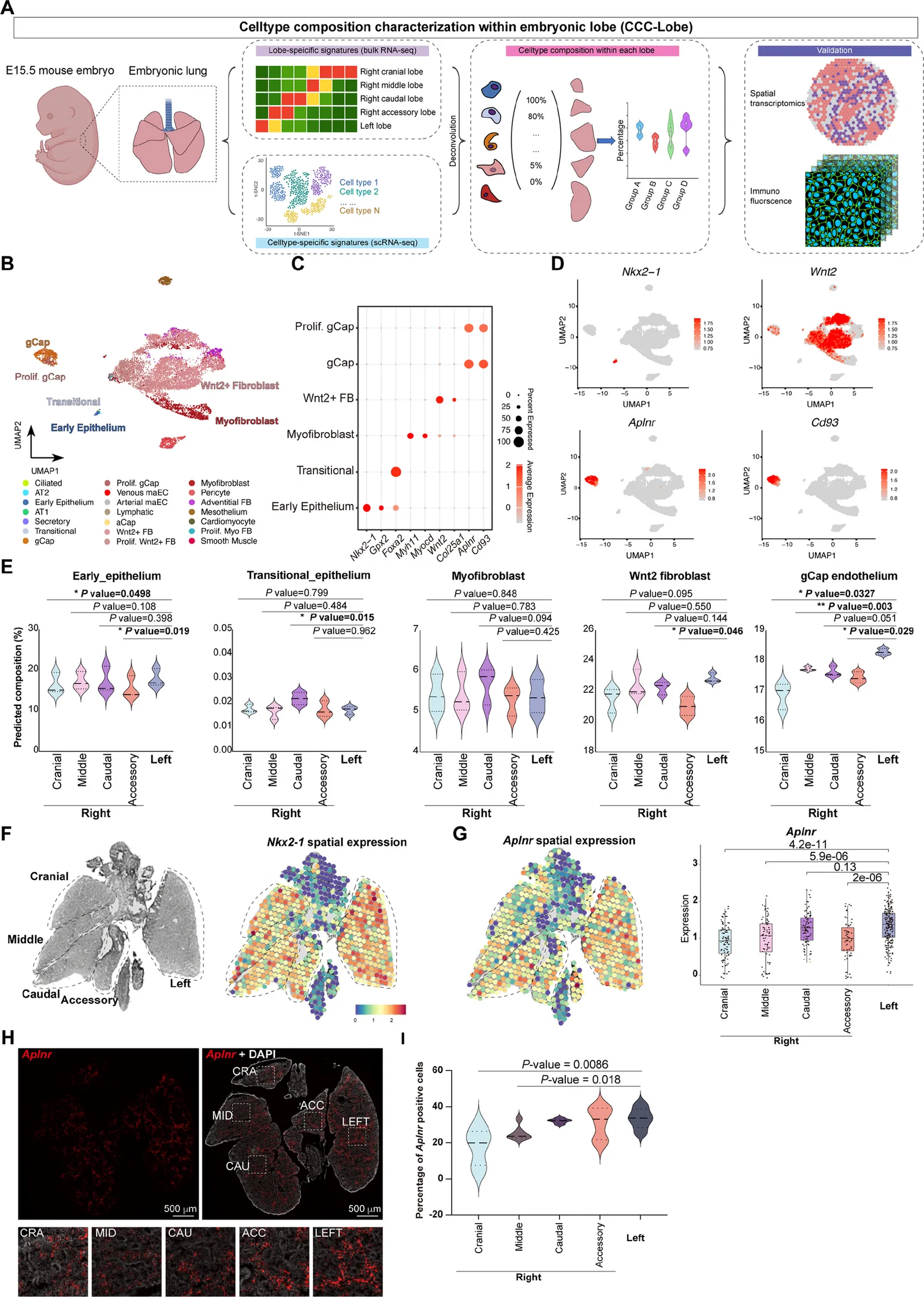
CCC-Lobe identifies a potential lobe-specific enrichment bias of gCap cells. **A** Schematic of the CCC-Lobe to predict the cell type compositions within each lobe. Lobe bulk transcriptome generated in this study was used for lobe registration; a stage-matched single cell transcriptomic dataset (Negretti et al. 2021) was used for cell type signature identification; a stage-matched spatial transcriptomic dataset (Meng et al. 2025) was used for cell type distribution validation. **B** UMAP projection of major cell types within E15.5 mouse embryonic lung based on single cell transcriptomic atlas (Negretti et al. 2021). Cells were colored based on previous annotation. **C** Dot plot (**C**) and feature plot (**D**) revealing the expression of signature genes across cell types as indicated. **E** Violin plot showing the predicted abundance for early epithelium, transitional epithelium, myofibroblast, Wnt fibroblast, and gCap endothelial cells across five lung lobes. **F** Original lung tissue morphology from the published spatial transcriptomic dataset (Meng et al. 2025) recording the lobe distribution. The expression of lung epithelium marker-*Nkx2-1* was shown. **G** Spatial distribution of *Aplnr* expression. The lobe-specific enrichment of *Aplnr* signal was calculated with statistics labelled. **H** RNAscope staining of *Aplnr* validating the lobe differential distributions of *Aplnr* expression. CRA: right cranial lobe; MID: right middle lobe; CAU: right caudal lobe; ACC: right accessory lobe; LEFT: left lobe. **I** Statistical summary of *Aplnr*-positive cell proportions across each lung lobe. Student’s t-test was used. Six cryosections were calculated

Although substantial transcriptional differences were identified across individual lobes, whether these regional signatures are accompanied by differences in cellular composition remains unclear. Integrative cell-type deconvolution approaches (Xu et al. 2024) that combine single-cell transcriptomic references with bulk RNA-seq data have been used to resolve complex tissue composition, such as the dissection of tumor microenvironment (Wang et al. 2026). Here, to probe whether the observed lobe-specific transcriptional signatures were associated with differences in cellular composition, we devised a framework of Cell type Composition Characterization resolved at Lobe resolution (CCC-Lobe) by integrating our in-depth lobe bulk transcriptomic dataset with published stage-match single-cell transcriptomic references from the embryonic lung (Fig. 4A). Generally, the specific cell types and relevant transcriptomic signatures were annotated and identified by referencing the published lung cell atlas (Negretti et al. 2021), including major subtypes of epithelium (early epithelium, transitional epithelium etc.), endothelium (proliferating gCap, gCap etc.), and mesenchyme cells (Wnt2 + fibroblast, myofibroblast etc.) (Fig. 4B). Representative marker genes expression further validated the inferred identities of these major cell states and related subtypes (Fig. 4C-D). For instance, *Nkx2-1* is expressed in epithelial cells, *Wnt2* is highly expressed in fibroblasts, and *Aplnr* and *Cd93* are specifically expressed in gCap cells (Fig. 4C-D). Then, we applied CCC-Lobe to predict the cell type abundance within each lobe (Fig. 4E). Surprisingly, we found that there do exist lobe-specific preferences for cell type abundance, especially for gCap cells. As shown, the left lobe shows the highest abundance of gCap endothelial cells, whereas the right cranial lobe shows the lowest. It has been reported that gCap cells can serve as a progenitor reservoir for repairing damaged capillaries (Gillich et al. 2020). The differential abundances of gCap cells across individual lobes indicate that lobe identity should be taken into consideration when evaluating the embryonic origin and developmental regulation of these specialized capillary progenitors, and also highlight the importance of examining lobe-specific responses during future potential vascular repair and regeneration.

To validate the lobe-specific pattern for cell type composition, we also took advantage of a recent publicly available spatial transcriptomic dataset (Meng et al. 2025), which consists of stage-matched spatial gene expression patterns for E15.5 embryonic lung (Fig. 4F). As expected, we found that the expression of the gCap marker—*Aplnr* also shows a consistent distribution pattern as predicted by CCC-Lobe, in which the left lobe shows the highest enrichment of *Aplnr* expression, and the right cranial lobe shows the least (Fig. 4G). Moreover, RNAscope staining of *Aplnr* transcripts further corroborates the inter-lobar heterogeneity of gCap cell distribution (Fig. 4H-I; Fig. S3A). Moreover, the observed molecular divergence for gCap endothelial cells may also have an A-P positional component in the left lobe, manifesting as relatively higher expression of *Cd93* and *Aplnr* in the middle part (Fig. S3B-C). Together, these data demonstrate that individual lung lobes heterogeneously harbor specific molecular and cellular signatures during embryonic development.

## Discussion

In the present study, we provide evidence that anatomically distinct lung lobes have already possessed measurable molecular and cellular heterogeneity during the pseudoglandular stage of mouse lung development. Although the global multi-lobar architecture is morphologically established at E15.5, our lobe-resolved transcriptomic analyses demonstrate that individual lobes are not developmentally equivalent. Instead, each lobe shows enrichment of specific gene expression modules together with reproducible distinction in cell type composition. These findings suggest that inter-lobar heterogeneity emerges during embryogenesis.

One notable observation is the preferential enrichment of immune-associated gene signatures within the left lobe. While the presence and roles of the immune microenvironment are largely unexplored, recent progress has reported that myeloid cells within the embryonic lung can promote lung epithelial progenitor exit from self-renewal and differentiation to basal cells by producing IL-1β (Barnes et al. 2023). Our data raise the possibility that the left lobe may possess an intrinsic predisposition toward immune responsiveness. Interestingly, a recent study reported that the left lobe shows stronger inflammatory responses following lipopolysaccharide stimulation (Yoshida et al. 2026). Though the left lobe-biased immune signatures identified in this study warrant further investigation, systematic characterization of the immune environment in embryonic and adult functional lung tissue will be important for a comprehensive understanding of its potential roles in pulmonary development and disease.

We also identified a relatively high enrichment of cardiac muscle morphogenesis related genes in the right cranial lobe. Given the anatomical proximity between the right cranial lobe and the developing heart, this pattern may reflect shared signaling, inter-organ interaction, or coordinated cardiopulmonary tissue patterning may arise during embryogenesis. Recently, it has been reported that the co-development of heart and lung is coordinated by multipotent cardiopulmonary progenitors (Peng et al. 2013). By combining both lineage tracing and single-cell sequencing, the *Wnt2*^+^ cardiopulmonary progenitors can contribute to nearly all lung mesoderm lineages (Wen et al. 2025). Based on these findings, integrative tracing of both cardiac and pulmonary lineages, together with specific cell types, will clarify whether the right cranial lobe could be a special developmental hotspot for cardiopulmonary progenitors’ differentiation relative to the other lobes.

We also developed a CCC-Lobe framework to dissect the cell type composition across the five lung lobes. In contrast to previous studies (Gillich et al. 2020), which show uniform distribution of gCap cells between right and left lobes, here we found that the gCap cells show strong inter-lobar variations at E15.5. These capillary cells have been implicated in vascular maintenance and regeneration in the postnatal lung; the higher abundance of gCap signatures in the left lobe may suggest that the generation of endothelial progenitors and following vascular specification may also possess lobe bias. Such regional gCap endothelial heterogeneity may also influence the development of vasculature and alveolar maturation, and potentially establish distinct regenerative niches in response to lung injury or pulmonary diseases, such as pulmonary fibrosis, pulmonary arterial hypertension. Future exploration of the mechanisms through incorporating more embryonic timepoints and clinical samples may provide important insights into regional vascular biology and shed new light on lung repair and regeneration.

Regional variations are also a well-recognized feature of the adult lung, with differences in gene expression, cellular composition, and tissue microenvironment (Firsova et al. 2025). Moreover, it has been found that multiple lung diseases also show lobe preference, especially for congenital lung diseases. For instance, bronchial atresia mostly affects the left upper lobe in humans, and congenital pulmonary airway malformations are more frequently observed in the lower lung lobe (Durhan et al. 2021). These regional variations and lobe-biased disease susceptibilities raise the possibility that lobe-specific signatures may be established, at least in part, during embryonic development. Thus, our study provides an initial clue toward understanding the potential developmental origins and future in-depth investigations will shed new light on the prevention and treatment of these severe pulmonary diseases.

Together, our findings establish a framework for studying the embryonic lung at lobar resolution and highlight the importance of considering spatial sub-organ organization in developmental studies. Future studies integrating temporal single-cell, spatial multi-omics, and functional perturbation approaches should help define how lobe-specific programs are established and how they influence lifelong pulmonary health and disease.

## Conclusions

Our findings reveal the embryonic emergence of inter-lobar molecular and cellular heterogeneity, which highlights the importance of considering spatial sub-organ organization in future pulmonary development and disease-related studies.

## Materials and methods

### Embryo collection and lung dissection

The wildtype C57/BL6J mouse was purchased from a local supplier. The mouse lung lineage tracing mouse line (B6J.129S6-Nkx2-1tm1Ikono/Mmjax) was acquired from the Jackson Laboratory. The collection and dissection of the mouse lung were performed following the published procedures (Del Moral and Warburton 2010) with minor adjustments. Briefly, timed-pregnant mice were sacrificed on post-coitum day 15. After removing the maternal skin and surrounding lipid tissues, the uterus containing the embryos was dissected out, rinsed thoroughly in ice-cold PBS to remove blood, and transferred to a fresh 10-cm Petri dish. Embryos were released by incising the uterine wall under a stereoscopic dissecting microscope. The embryos were harvested using a perforated spoon and transferred to a new 10-cm petri-dish with iced PBS buffer.

For lung tissue dissection, each embryo was placed on its left flank. The right flank of the embryo, from the abdomen to the neck area, was opened using fine forceps. The heart and overlying tissues were carefully removed. The lung should be observable, lying posterior to the heart and anterior to the spine. Carefully removed the surrounding tissues around the lung and the trachea before isolating the embryonic lungs. Washed the lung tissue twice with PBS to remove residual tissues or blood. The acquired lungs were then subjected to further micro-dissection to collect each lung lobe.

### RNA extraction and whole transcriptome library preparation

Lung lobe tissues were first cut into small pieces and then added into the TRIzol reagent (Shanghai Pufei Biotechnology) (Yang et al. 2018) for thorough dissolution. Mixed vigorously to fully lyse the cells, and then incubated the lysate at room temperature for 5 min. Total RNA was extracted following chloroform separation and isopropanol precipitation, and finally resolved in nuclease free water.

500 ng total RNA was used for ribosomal RNA depletion using a commercial kit (Vazyme, N406). The removal of ribosomal RNA was performed following the manufacturer’s instructions. The purified total RNA was then subjected to transcriptome sequencing library preparation using an RNA-seq library Prep Kit for Illumina (Vazyme, NR606). The acquired RNA-seq libraries were finally sequenced with an Illumina NovaSeq 6000 (Annoroad gene technology).

### RNAscope and QuPath analysis

OCT-embedded E15.5 mouse embryo lungs were cryo-sectioned at 12 μm thickness serially from the ventral to dorsal region and mounted on electrostatic glass slides. Analysis of gene expression was performed using RNAscope® Multiplex Fluorescent Reagent Kit v2 (Advanced Cell Diagnostics, 323100) following manufacturer’s instructions with minor modifications (proteinase plus treatment for 15 min instead of proteinase IV for 30 min, and an additional 2 h photochemical bleaching step after probe hybridization using probes supplied by Advanced Cell Diagnostics: Mm-Aplnr-C3 (436171-C3). Images were acquired using the SLIDEVIEW VS200. QuPath was used to identify nuclei based on DAPI staining. Subsequently, a cell classifier was used to quantify *Aplnr*-positive cells and export the data.

### RNA-seq data processing and analyses

#### Sequencing data quality control and data processing

Data quality control was performed to ensure the reliability of the results. Quality control metrics such as sequence quality scores, GC content, and adapter content were assessed using FastQC. Low-quality reads and adapters were trimmed or removed by Trim Galore. Then, the high-quality reads were aligned to the GRCm39 reference genome using Hisat2. We assembled and quantified RNA-Seq transcripts using StringTie with default parameters. For quantification of the gene expression matrix, we used the Python script provided with StringTie, and then the count matrix was normalized to account for differences in sequencing depth and gene length as Transcripts Per Kilobase of exon model per Million mapped reads (TPM).

We loaded the GRCm39 annotation GTF file and extracted lncRNA and mRNA entries according to their gene biotypes. Expression matrices for lncRNAs and mRNAs were then generated separately for downstream differential analysis.

#### Principal component analysis

For the lncRNA and mRNA expression tables, we calculated the variance of each expressed gene across all samples and selected the top 1,000 most variable genes as highly variable genes (HVGs) for principal component analysis (PCA) using the R package FactoMineR.

#### Differentially expressed genes analysis

Differential expression analysis was performed using the R package limma. Pairwise comparisons were conducted between different lung lobes, treating each lobe as a distinct group. Genes (including both protein-coding mRNAs and long non-coding RNAs) were defined as differentially expressed genes (DEGs) if they met the following criteria: an adjusted *p*-value (FDR) < 0.05 and an absolute log₂ fold change > 0.5. The resulting DEGs were visualized as a heatmap using the R package ComplexHeatmap. Specific DEGs were validated through quantitative PCR (Table S2) and immunofluorescence (GATA4, Abcam, AB307823).

#### Functional enrichment analysis

We performed GO enrichment analysis on the differentially expressed genes (DEGs) using the R package clusterProfiler. Significantly enriched GO terms were identified using a one-sided hypergeometric test with Benjamini–Hochberg (BH) correction for multiple testing and were subsequently visualized.

#### Cell type composition deconvolution

To characterize the cellular composition across distinct lung lobes, we downloaded a publicly available mouse lung (E15) single-cell RNA sequencing (scRNA-seq) dataset as a reference and performed deconvolution of our bulk RNA-seq data using R-based deconvolution package BayesPrism. To enhance the signal-to-noise ratio of the deconvolution, we constructed the feature matrix using marker genes that were differentially and specifically upregulated in each major cell type.

## Supplementary Information

Supplementary Material 1. Fig. S1 Quality control for transcriptomic dataset. Fig. S2 Identification of differentially expressed long non-coding RNAs. Fig. S3 Quantification of *Aplnr*-postivie cells across each lobe.

Supplementary Material 2. Table. S1 Differentially expressed genes among distinct lobes.

Supplementary Material 3. Table. S2 Oligos used in this study.

## Data Availability

The raw sequencing dataset of whole transcriptome for lung lobes was deposited in the Genome Sequence Archive in National Genomics Data Center (CNCB-NGDC Members and Partners, 2024), China National Center for Bioinformation/Beijing Institute of Genomics, Chinese Academy of Sciences (GSA: CRA042367), which is publicly accessible at https://ngdc.cncb.ac.cn/gsa. Any other materials related to this study will be available from the corresponding author upon reasonable request.

## Abbreviations

E: Embryonic day
gCap: General capillary
rRNA: Ribosomal RNA
LncRNA: Long non-coding RNA
LPS: Lipopolysaccharide
PCA: Principal component analysis
HVGs: Highly variable genes
CCC-Lobe: Cell type Composition Characterization resolved at Lobe resolution
DEGs: Differentially expressed genes

## References

[CR1] Barnes JL, Yoshida M, He P, Worlock KB, Lindeboom RGH, Suo C, et al. Early human lung immune cell development and its role in epithelial cell fate. Sci Immunol. 2023;8(90):eadf9988. 10.1126/sciimmunol.adf9988. PMC7615868 38100545

[CR2] Del Moral PM, Warburton D. Explant culture of mouse embryonic whole lung, isolated epithelium, or mesenchyme under chemically defined conditions as a system to evaluate the molecular mechanism of branching morphogenesis and cellular differentiation. Methods Mol Biol. 2010;633:71–9. 10.1007/978-1-59745-019-5_5. PMC3120103 20204620

[CR3] Durhan G, Ardali Duzgun S, Akpinar MG, Demirkazik F, Ariyurek OM. Imaging of congenital lung diseases presenting in the adulthood: a pictorial review. Insights Imaging. 2021;12(1):153. 10.1186/s13244-021-01095-2. PMC8557233 34716817

[CR4] Firsova AB, Marco Salas S, Kuemmerle LB, Abalo XM, Sountoulidis A, Larsson L, et al. Spatial single-cell atlas reveals regional variations in healthy and diseased human lung. Nat Commun. 2025;16(1):9745. 10.1038/s41467-025-65704-0. PMC12589588 41193468

[CR5] Gillich A, Zhang F, Farmer CG, Travaglini KJ, Tan SY, Gu M, et al. Capillary cell-type specialization in the alveolus. Nature. 2020;586(7831):785–9. 10.1038/s41586-020-2822-7. PMC7721049 33057196

[CR6] Han L, Chaturvedi P, Kishimoto K, Koike H, Nasr T, Iwasawa K, et al. Single cell transcriptomics identifies a signaling network coordinating endoderm and mesoderm diversification during foregut organogenesis. Nat Commun. 2020;11(1):4158. 10.1038/s41467-020-17968-x. PMC7453027 32855417

[CR7] Ikonomou L, Herriges MJ, Lewandowski SL, Marsland R 3rd, Villacorta-Martin C, Caballero IS, et al. The in vivo genetic program of murine primordial lung epithelial progenitors. Nat Commun. 2020;11(1):635. 10.1038/s41467-020-14348-3. PMC6994558 32005814

[CR8] Krogh A. The comparative physiology of respiratory mechanisms. University of Pennsylvania press; 2015. 10.2307/j.ctv5rdxfn.

[CR9] Longmire TA, Ikonomou L, Hawkins F, Christodoulou C, Cao Y, Jean JC, et al. Efficient derivation of purified lung and thyroid progenitors from embryonic stem cells. Cell Stem Cell. 2012;10(4):398–411. 10.1016/j.stem.2012.01.019. PMC3322392 22482505

[CR10] Meng X, Li W, Xu J, Yao Y, Gong A, Yang Y, et al. Spatiotemporal transcriptome atlas of developing mouse lung. Sci Bull (Beijing). 2025;70(10):1641–58. 10.1016/j.scib.2025.03.012. 40118721

[CR11] Metzger RJ, Klein OD, Martin GR, Krasnow MA. The branching program of mouse lung development. Nature. 2010;453(7196):745–50. 10.1038/nature07005. PMC2892995 18463632

[CR12] Miao Y, Pek NM, Tan C, Jiang C, Yu Z, Iwasawa K, et al. Co-development of mesoderm and endoderm enables organotypic vascularization in lung and gut organoids. Cell. 2025;188(16):4295-313 e27. 10.1016/j.cell.2025.05.041. PMC13056595 40592324

[CR13] Negretti NM, Plosa EJ, Benjamin JT, Schuler BA, Habermann AC, Jetter CS, et al. A single-cell atlas of mouse lung development. Development. 2021;148(24). 10.1242/dev.199512. PMC8722390 34927678

[CR14] Ng WH, Varghese B, Jia H, Ren X. Alliance of heart and endoderm: multilineage organoids to model co-development. Circ Res. 2023;132(4):511–8. 10.1161/CIRCRESAHA.122.321769. PMC9942098 36795851

[CR15] Peng T, Tian Y, Boogerd CJ, Lu MM, Kadzik RS, Stewart KM, et al. Coordination of heart and lung co-development by a multipotent cardiopulmonary progenitor. Nature. 2013;500(7464):589–92. 10.1038/nature12358. PMC3758448 23873040

[CR16] Schittny JC. Development of the lung. Cell Tissue Res. 2017;367:427–44. 10.1007/s00441-016-2545-0. PMC5320013 28144783

[CR17] Wang Y, Hu W, Xia R, Yang X, Gu Y, Ning Z, et al. CLIM-TIME identifies metastatic microenvironment modulators for T cell therapy response. Cell. 2026;189(5):1555-72 e23. 10.1016/j.cell.2025.12.042. 41679300

[CR18] Wen H, Chandrasekaran P, Jin A, Pankin J, Lu M, Liberti DC, et al. A spatiotemporal cell atlas of cardiopulmonary progenitor cell allocation during development. Cell Rep. 2025;44(4):115513. 10.1016/j.celrep.2025.115513. PMC12103214 40178979

[CR19] Xu X, Li R, Mo O, Liu K, Li J, Hao P. Cell-type deconvolution for bulk RNA-seq data using single-cell reference: a comparative analysis and recommendation guideline. Brief Bioinform. 2024;26(1). 10.1093/bib/bbaf031. PMC11789683 39899596

[CR20] Yang X, Wang R, Wang X, Cai G, Qian Y, Feng S, et al. TGFbeta signaling hyperactivation-induced tumorigenicity during the derivation of neural progenitors from mouse ESCs. J Mol Cell Biol. 2018;10(3):216–28. 10.1093/jmcb/mjy013. 29481611

[CR21] Yang X, Chen Y, Yang Y, Li S, Mi P, Jing N. The molecular and cellular choreography of early mammalian lung development. Med Rev (2021). 2024;4(3):192–206. 10.1515/mr-2023-0064. PMC11195428 38919401

[CR22] Yoshida SY, Matsumoto K, Takagi S, Kinoshita FL, Yamashita K, Shigeta D, et al. Whole-organ and whole-body 3D atlases enable cellome-wide profiling. Cell. 2026;189(6):1836-53 e19. 10.1016/j.cell.2025.12.057. 41747726

[CR23] Zepp JA, Morley MP, Loebel C, Kremp MM, Chaudhry FN, Basil MC, et al. Genomic, epigenomic, and biophysical cues controlling the emergence of the lung alveolus. Science. 2021;371(6534). 10.1126/science.abc3172. PMC8320017 33707239

