# Supplementary material for: Early emergence of inter-lobar heterogeneity in mouse embryonic lung
Source: Cell Regen. 2026 Oct 14;15:31. doi: 10.1186/s13619-026-00303-y (PMC13669285; doi:10.1186/s13619-026-00303-y)
Supplement: Supplementary file 1 [file 13619_2026_303_MOESM1_ESM.pdf]

Supplementary Fig. S1 Quality control for transcriptomic dataset.
A. Bar plots showing the numbers of mRNAs detected across every lobe.
B. Bar plots showing the numbers of lncRNAs detected across each lobe.
C. Scatter plot showing the transcriptomic correlation between biological replicates for each lobe.

Supplementary Fig. S2 Identification of differentially expressed long non-coding RNAs.
A. Heatmap showing the lobe-specific lncRNA expression patterns. Differentially expressed genes were identified based on the criteria: adjusted p-value (FDR) <0.05, an absolute $\log_2$(fold_change) > 0.5.
B. Line plots displaying the scaled expression level of lobe-specific lncRNAs.
C. The expression of cardiomyocyte markers (*Myl2*, *Myl4*, *Nkx2-5*) in heart and dissected lung lobes.

Supplementary Fig. S3 Quantification of *Aplnr*-postivie cells across each lobe.
A. Representative images recording the quantification of *Aplnr*-positive cells with QuPath.
B. The division of the left lobe into anterior, middle, and posterior sections.
C. The expression of *Cd93* and *Aplnr* along the A-P axis in the left lobe. Wilcox tests were used to calculate the significance.

Supplementary Table S1. Differentially expressed genes among distinct lobes.

Supplementary Table S2. Oligos used in this study.

Fig. S1

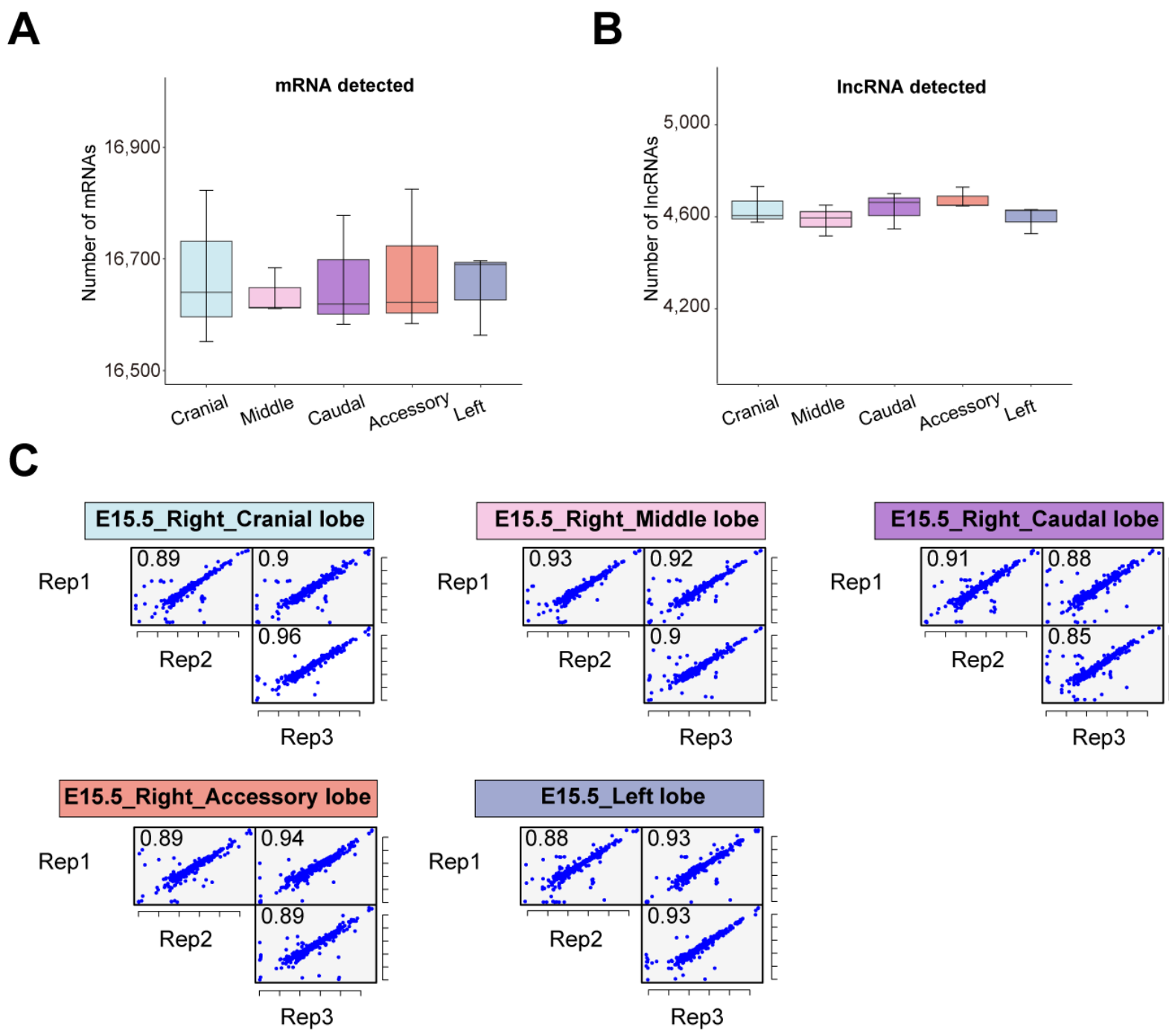
A
mRNA detected
Number of mRNAs
16,900
16,700
16,500
Cranial
Middle
Caudal
Accessory
Left
B
lncRNA detected
Number of lncRNAs
5,000
4,600
4,200
Cranial
Middle
Caudal
Accessory
Left
C
E15.5_Right_Cranial lobe
Rep1
0.89
0.9
0.96
Rep2
Rep3
E15.5_Right_Middle lobe
Rep1
0.93
0.92
0.9
Rep2
Rep3
E15.5_Right_Caudal lobe
Rep1
0.91
0.88
0.85
Rep2
Rep3
E15.5_Right_Accessory lobe
Rep1
0.89
0.94
0.89
Rep2
Rep3
E15.5_Left lobe
Rep1
0.88
0.93
0.93
Rep2
Rep3

Fig. S2

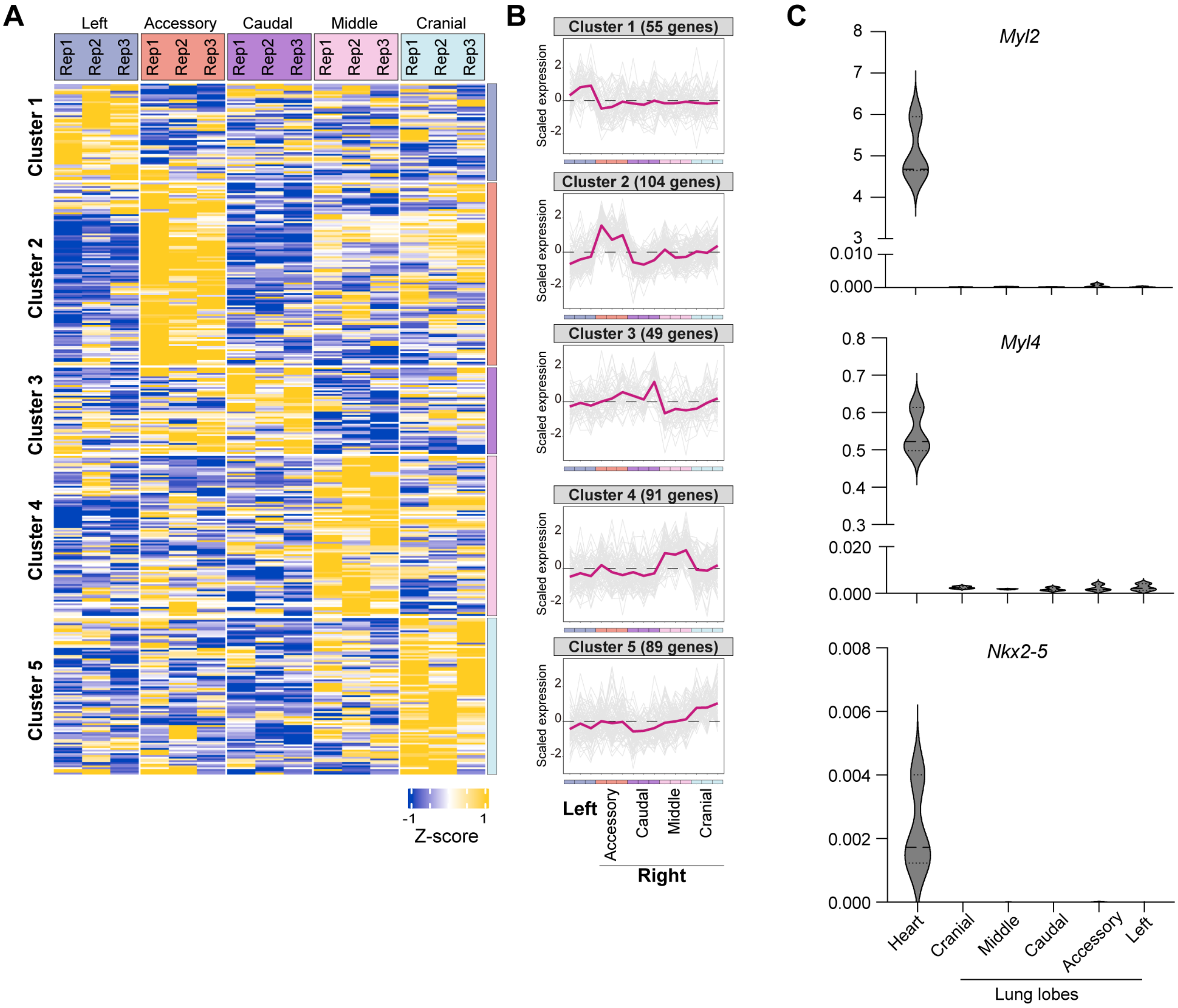
A
Left
Accessory
Caudal
Middle
Cranial
Rep1
Rep2
Rep3
Cluster 1
Cluster 2
Cluster 3
Cluster 4
Cluster 5
-1
1
Z-score
B
Cluster 1 (55 genes)
Cluster 2 (104 genes)
Cluster 3 (49 genes)
Cluster 4 (91 genes)
Cluster 5 (89 genes)
Scaled expression
Left
Accessory
Caudal
Middle
Cranial
Right
C
Myl2
Myl4
Nkx2-5
Heart
Cranial
Middle
Caudal
Accessory
Left
Lung lobes

Fig. S3

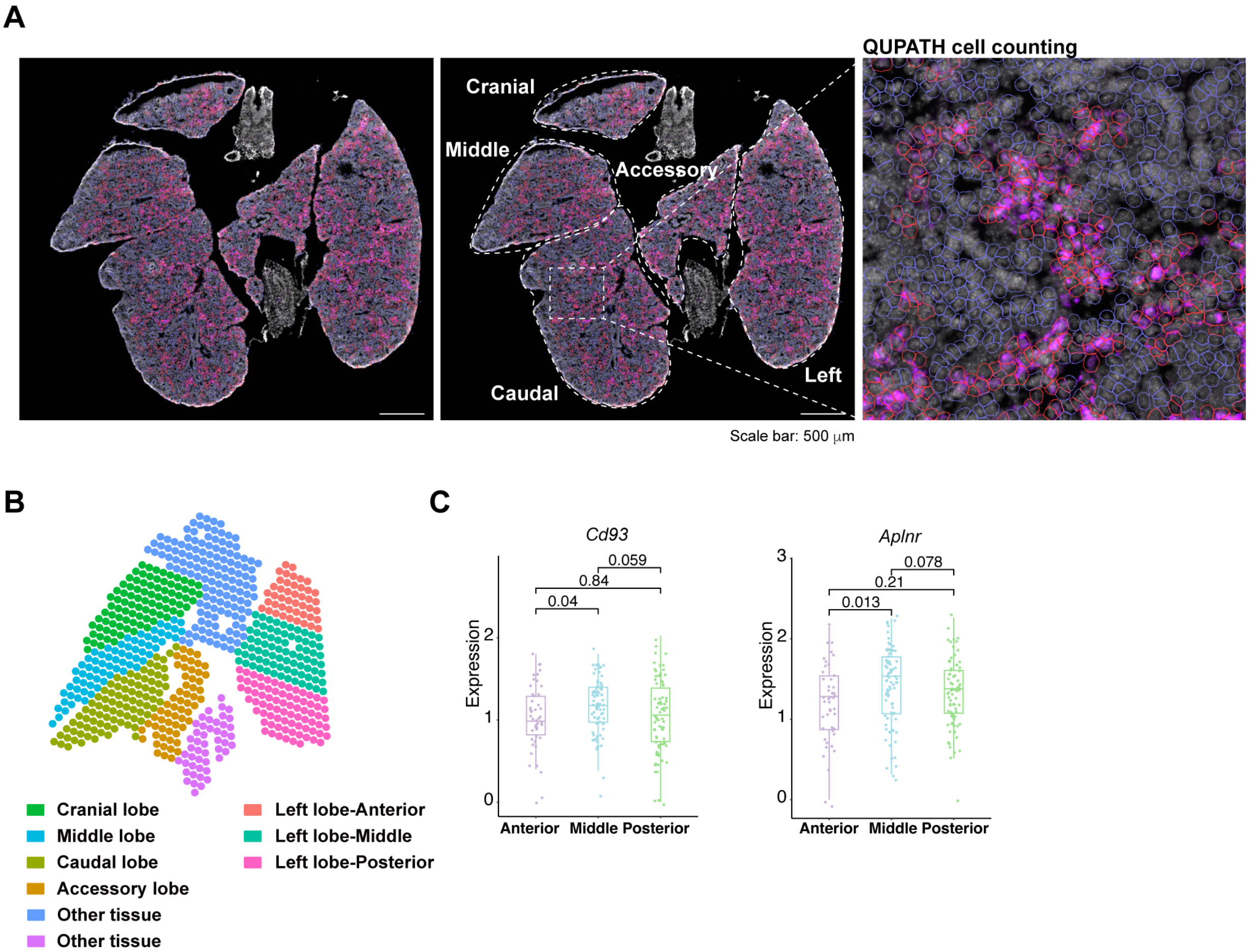
A
QUPATH cell counting
Cranial
Middle
Accessory
Caudal
Left
Scale bar: 500 µm
B
Cranial lobe
Middle lobe
Caudal lobe
Accessory lobe
Other tissue
Other tissue
Left lobe-Anterior
Left lobe-Middle
Left lobe-Posterior
C
Cd93
Aplnr
Expression
0.059
0.84
0.04
0.078
0.21
0.013
Anterior
Middle
Posterior
